# Recent progress in the development of etomidate analogues

**DOI:** 10.3389/fphar.2025.1614865

**Published:** 2025-10-14

**Authors:** Yanting Chen, Lan Wu, Bingchen Lang, Wensheng Zhang, Shouming Chen

**Affiliations:** ^1^ Department of Anesthesiology, West China Second University Hospital, Sichuan University, Chengdu, Sichuan, China; ^2^ Key Laboratory of Birth Defects and Related Diseases of Women and Children (Sichuan University), Ministry of Education, Chengdu, Sichuan, China; ^3^ West China School of Medicine, Sichuan University, Chengdu, Sichuan, China; ^4^ Department of Pharmacy, West China Second University Hospital, Sichuan University, Chengdu, Sichuan, China; ^5^ Department of Anesthesiology, West China Hospital, Sichuan University, Chengdu, Sichuan, China; ^6^ Laboratory of Anesthesia and Critical Care Medicine, National-Local Joint Engineering Research Centre of Translational Medicine of Anesthesiology, West China Hospital, Sichuan University, Chengdu, Sichuan, China

**Keywords:** etomidate, etomidate analogues, CPMM, ET-26HCl, MOC-etomidate

## Abstract

Etomidate is a widely utilized anaesthetic agent for the induction of general anesthesia, recognized for its rapid onset and minimal hemodynamic suppression effects. However, its clinical application is constrained by several adverse effects, including adrenal cortex suppression, postoperative nausea and vomiting, and myoclonus. In recent years, there has been a growing global research focus on structural modifications of the etomidate molecule, aiming to simultaneously ameliorate its adverse effects and optimize its hemodynamic stability efficacy. Methoxycarbonyl-etomidate and carboetomidate are inhibited due to the accumulation of their metabolites. CPMM and ET-26HCI have entered the clinical trial stage, but there are still adverse reactions and challenges for the next phase of research. This comprehensive review systematically examines recent scientific advancements in this field, focusing on structural modifications, pharmacological properties, and clinical translation of these novel compounds.

## 1 Background

Etomidate is a positive allosteric modulator (PAM) based on an imidazole structure, which chiefly enhances γ-aminobutyric acid type A (GABA_A_) effects only at high concentrations (Philip et al., 2025). As an intravenous anesthetic, it exhibits a rapid onset (15–20 s), a short elimination half-life (2.9–5.5 h), and a high therapeutic index (26.4) (Janssen et al., 1975; Kay, 1976; Zed et al., 2006; Zhang et al., 2024; Group, 2024). Patients typically experience quick recovery times (5–15 min) after continuous infusion or repeated administration, making it widely used for anesthesia induction and maintenance (Taiyun Wang and Li, 2021; Ding et al., 2024; van den Heuvel et al., 2013). Notably, one of etomidate’s most significant advantage is its stable hemodynamics, making it the preferred choice for anesthesia induction in critically ill patients with unstable hemodynamics, such as those undergoing cardiovascular surgery or suffering from hemorrhagic shock (Forman, 2011; Chan et al., 2012). Additionally, studies have demonstrated that etomidate significantly reduces the risk of respiratory depression and cerebral ischemia-reperfusion injury (Jung et al., 2020; Deng Hong et al., 2017). Considering the age-related decline in physiological reserve, etomidate also represents an optimal anesthetic selection for geriatric patients, owing to its minimal cardiovascular depressant effects and predictable pharmacokinetic profile (Taiyun Wang and Li, 2021; Egan and Johnson, 2020).

The clinical administration of etomidate is associated with several well-documented adverse effects, including injection site pain, transient myoclonic movements, postoperative nausea and vomiting (PONV), and adrenocortical suppression, which represents its most significant pharmacological limitation (Yelavich and Holmes, 1980; Meinck et al., 1980; De Jong and Jaber, 2014). Among these adverse effects, the adrenocortical suppression has emerged as a particular focus of scientific investigation (Ledingham and Watt, 1983; Wagner et al., 1984; Allolio et al., 1984; Srivilaithon et al., 2023). Since the study by Ledingham and colleagues (Ledingham and Watt, 1983; Wagner et al., 1984; Allolio et al., 1984) in 1983 demonstrated an association between etomidate infusion and increased mortality rates in critically ill patients, the clinical application of etomidate has been substantially limited, particularly in the context of continuous or prolonged administration. Subsequent studies have shown that etomidate inhibits the activity of 11-β-hydroxylase and 17-α-hydroxylase, leading to reduced cortisol production and adrenal insufficiency, making it unsuitable for prolonged anesthesia maintenance or sedation (Wagner et al., 1984; Allolio et al., 1984).

While the mechanisms underlying etomidate-induced adrenocortical suppression have been partially characterized, the relationship between etomidate administration and mortality in critically ill patients remains inconclusive. Emerging clinical evidence indicates that even a single bolus dose of etomidate for anesthesia induction may induce transient adrenocortical suppression lasting up to 72 h in approximately 7% of patients with sepsis or septic shock (Vinclair et al., 2008). Furthermore, the investigation by Komatsu et al. (2013) revealed that etomidate administration was associated with significantly worse clinical outcomes, including elevated mortality rates, increased cardiovascular complications, and prolonged hospitalization durations when compared to both volatile anesthetic agents and propofol-based anesthesia. However, contrasting perspectives from a another research group maintain that although single-dose etomidate administration may elevate the risk of transient adrenal insufficiency in septic patients, current evidence fails to establish a definitive causal relationship with increased mortality rates (Gu et al., 2015). The inconsistent findings across studies likely reflect the complex interplay of surgical factors, baseline of patient characteristics, and methodological challenges inherent in critical care research, such as heterogeneity in disease severity and treatment protocols.

In order to determine the relationship between etomidate administration and mortality rate, well-designed randomized controlled trials are needed (Chen Shoushou, 2016). However, due to ethical, financial, and efficiency concerns (Chen Shoushou, 2016), research efforts have predominantly shifted toward structural modification of the etomidate molecule to develop new analogues that retain its hemodynamic stability while eliminating adrenocortical suppression (Egan, 2009; Liu et al., 2024). The main characteristics of etomidate derivatives mentioned in the following article are summarized in Table 1.

**TABLE 1:** Summary of the characteristics of Etomidate’s main derivatives.

	MOC-etomidate	Carboetomidate	CPMM	ET-26HCl	EL-0052	ET-25–2	ET-11B
Adrenal Suppression	Relative reduction vs. etomidate	∼1000× reduced vs. etomidate	Significantly reduced vs. etomidate	Significantly reduced vs. etomidate	Relative reduction vs. etomidate	Relative reduction vs. etomidate	Relative reduction vs. etomidate
Hemodynamic Stability	Hemodynamically stable as etomidate	Hemodynamically stable as etomidate	Hemodynamically stable as etomidate	Hemodynamically stable as etomidate	Hemodynamically stable as etomidate	Hemodynamically stable as etomidate	Hemodynamically stable as etomidate
Side effects	Metabolite accumulation	Metabolite accumulation	Involuntary muscle movements (IMM)	Myoclonus, Bradycardia	Unclear	Unclear	Unclear
Preclinical animal studies	Single administration: half-life is 4.4 min in rat	Single administration: The cortisol reduction was 1/1000 that of etomidate in rat	Single administration: Compared with etomidate, CPMM was half as potent as a hypnotic (ED_50_: 0.8 mg kg^-1^) in beagle dog, and had a shorter duration of sedative-hypnotic action Recovery time after CPMM administration were independent of infusion duration	Single administration: Stable hemodynamics and mild adrenal suppression, with faster recovery of spatial orientation compared to propofol in rat	Single administration The TI of EL-0052 in rats was 28, which was higher than 22 of etomidate There was no significant difference in hypnotic onset time, recovery time, and walking time compared with etomidate in rats EL-0052 had no signifi can’t effect on adrenocortical function in dogs even at a high dose (4.3 × ED_50_) EL-0052 had a weak inhibition on cortisol biosynthesis in human H259 cells with an IC_50_ of 1050 ± 100 nM, which was 2.09 ± 0.27 nM for etomidate	ET-25–2 retains the advantage of quick onset of etomidate. The maintenance time of anesthesia was shorter (P ≤ 0.05)., it showed no adrenal suppression in rats	Single administration: ET-11B compared with etomidate (ED_50_:1.46 mg/kg vs. 0.74 mg/kg) in rat ET-11B onset time less than 1 min in aged rat
			Continuous infusion: CPMM and etomidate achieved 80% EEG burst suppression for 120 min, with total doses of 143 mg kg^-1^ and 36 mg kg^-1^,cortisol levels in the CPMM rat group returned to normal within 30 min, while the etomidate group did not recover even after 3 h	ET-26HCl has a lower minimum infusion rate in rats compared to CPMM (0.62 mg kg^-1^·h^-1^ vs. 0.95 mg kg^-1^·h^-1^)			ET-11B and saline groups showed no significant differences in serum corticosterone levels or changes from baseline at any time point after ACTH administration (P > 0.05)
	Continuous Infusion: The metabolite MOC-ECA has sedative activity, prolong recovery time and cause EEG burst suppression in rat	Repeated administration in rat: Mild cortisol suppression; reduce release of inflammatory factors	Rapid Metabolism: with no significant accumulation of metabolites in rat	Continuous infusion: 0.62 mg kg^-1^·h^-1^ for 60 min did not cause significant adrenal suppression in rat	Continuous infusion: did not conduct the hypnotic tests The pharmacokinetic properties of EL-0052 were not explored yet.		Under basic anesthesia, ET-11B decrease HR was less than 5% in rat with hemorrhagic shock, while etomidate greater than 10%
			Reduce inflammatory factors and mortality rates compared with etomidate in lipopolysaccharide-induced septic rat model	Reduced plasma cytokine levels Less histological damage to the lungs and kidneys in rat			
Phase I clinical trial	None Accumulation of MOC-ECA may lead to delayed recovery	None Accumulation of MOC-ECA may lead to delayed recovery	Well-tolerated at a single dose of up to 1.0 mg kg^-1^, optimal recommended dose for anesthesia induction determined to be 0.25–0.35 mg kg^-1^ in healthy nonsmoking men and women aged between 18 and 45 years with a BMI between 17.5 and 30 kg/m^2^	Single dose of up to 2.8 mg kg^-1^: Larger area under curve of plasma total cortisol (AUC_PTC_) compared to 0.3 mg kg^-1^ etomidate, indicating milder adrenal suppression in healthy men and women aged 18–45 years, with a BMI 19.0–24.0 kg/m^2^	None	None The titer of ET-25–2 is relatively low and it is difficult to prepare salts with sufficient water solubility	None
			Alone or in combination with commonly used adjuncts: Efficacy is not affected by preoperative or combination medications in healthy men and women aged between 18 and 53 years				
			Continuous infusion: At 30, 40, and 50 μg kg^-1^ min^-1^ for 30 min, did not cause clinically significant adrenal suppression in healthy (ASA physical status I), non-smoking men and women aged 18–45 years In this study, incidence of IMM >20%, and some are accompanied by activities similar to epilepsy				
Phase II clinical trial	None	None	None Incidence of IMM is high	The induction success rate of tracheal intubation within 7 min after induction with 0.8 mg kg^-1^ is 100%, MOAA/S scores ≤1, and no myoclonus was observed in healthy (ASA physical status 1–2) patients aged 18–65 years, with a BMI between 18 and 30 kg/m^2^	None	None	None

## 2 Methoxycarbonyl-etomidate

Methoxycarbonyl-etomidate (MOC-etomidate) is designed similarly to remifentanil and esmolol, with a diester bond added to the distal end of the ester bond in etomidate to form a metabolically unstable ester group (Ge et al., 2013). This structural modification allows MOC-etomidate rapid hydrolysis into a less active methoxycarbonyl-etomidate carboxylic acid (MOC-ECA), which reducing adrenal suppression to nearly 1/400 of that of MOC-etomidate (Chitilian et al., 2013; Husain et al., 2012), while retaining or even improving its hemodynamic stability (Cotten et al., 2009). Pejo et al. (2012a) found that MOC-etomidate is rapidly metabolized in rats, with a half-life of only 4.4 min. However, prolonged infusion of high doses of MOC-etomidate increased the recovery time of the righting reflex in rats, and the recovery speed of the electroencephalogram (EEG) was positively correlated with the infusion time (Pejo et al., 2012b). This phenomenon may be attributed to the accumulation of MOC-ECA, which retains sedative properties and may even induce EEG burst suppression. Given the rapid metabolic clearance of MOC-etomidate observed in rats, high doses are required to maintain adequate sedation, and potentially leading to significant accumulation of MOC-ECA, an active metabolite. This pharmacokinetic profile raises particular concerns, as the MOC-ECA’s sedative properties and potentially accumulation in patients with renal insufficiency (Zhang Jingwen, 2019), making it less ideal for critically ill patients. While these pharmacological limitations have hindered further development of MOC-etomidate, but as the first designed soft drug etomidate analogue, it has provided valuable insights for subsequent drug development.

## 3 Carboetomidate

Etomidate inhibits 11-β-hydroxylase, preventing the conversion of 11-deoxycortisol to cortisol (Fry and Griffiths, 1984; Fragen et al., 1984). Additionally, its mild inhibition of 17-α-hydroxylase leads to increased levels of cortisol precursors such as 11-deoxycortisol, 17-hydroxyprogesterone, and adrenocorticotropic hormone (ACTH), adversely affecting the endocrine system (Pan, 2013; de Jong et al., 1984; Wagner and White, 1984). To explore the high affinity of etomidate for 11-β-hydroxylase, studies using homology modeling revealed that the nitrogen atom on the imidazole ring of etomidate binds to the heme iron in the enzyme’s active center. Based on this, carboetomidate was developed by replacing this nitrogen atom with a methylene group, reducing the inhibition of 11-β-hydroxylase and minimizing the impact on cortisol synthesis (Chitilian et al., 2013; Pejo et al., 2012c; Roumen et al., 2007). Compared to etomidate, carboetomidate reduces adrenal suppression by approximately 1000-fold (Cotten et al., 2010), shows milder cortisol suppression in septic rat models, and releases fewer inflammatory cytokines (Pejo et al., 2012c), maintaining immune regulation. Even after repeated administration high doses, carboetomidate can be beneficial in critical care settings of sepsis rats after injection of lipopolysaccharide (Pejo et al., 2012c). Additionally, carboetomidate reduces the incidence of POVN by inhibiting and accelerating the inactivation of 5-HT3A receptors (Desai et al., 2013). However, carboetomidate, similar to MOC-etomidate, is easily hydrolyzed by esterases into the carboxylic acid metabolite MOC-ECA. Given the adverse effects of MOC-ECA (Zhang Jingwen, 2019), further research on carboetomidate also has been limited (Philip et al., 2025).

## 4 Cyclopropyl-methoxycarbonyl-metomidate

The development of cyclopropyl-methoxycarbonyl-metomidate (CPMM, ABP-700) was motivated by MOC-etomidate’s short metabolic half-life (4.4 min) (Pejo et al., 2012a) and the anesthetic properties of its MOC-ECA metabolite (Zhang Jingwen, 2019), aiming to address the delayed recovery from anesthesia caused by prolonged use of MOC-etomidate and to extend its metabolic and action duration (Husain et al., 2012; Bodor and Buchwald, 2000). In rat models, continuous infusion of CPMM and etomidate achieved 80% EEG burst suppression for 120 min, with total doses of 143 mg kg^-1^ and 36 mg kg^-1^, respectively (Pejo et al., 2012b; Ge et al., 2013). After infusion, cortisol levels in the CPMM rat group returned to normal within 30 min, while the etomidate group did not recover even after 3 h, this suggests that CPMM exhibits attenuated adrenocortical suppression, and potentially attributable to its characteristic rapid onset of action and swift metabolic clearance (Pejo et al., 2012b). Recent studies showed that CPMM had similar adrenal cortical reactivity to propofol in beagle dogs 90 min after injection, while exhibiting significantly enhanced anesthetic efficacy relative to MOC-etomidate (Husain et al., 2012). Continuous infusion CPMM in rats for 2 h did not show significant accumulation of carboxylic acid metabolites in blood and cerebrospinal fluid (Pejo et al., 2016). Additionally, in a lipopolysaccharide-induced septic rat model, CPMM reduced plasma cytokine levels (IL-1β, IL-6, and IL-10) within 1 h and had lower mortality compared to etomidate group rat (Santer et al., 2015). Owing to its ability to be prolonged infusion and its favorable pharmacological properties in septic models, CPMM has advanced to human clinical trials (Khurmi et al., 2017).

In a 2017 single-center, double-blind, placebo-controlled Phase I clinical trial (Struys et al., 2017), CPMM was well-tolerated at a single dose of up to 1.0 mg kg^-1^, with the optimal recommended dose for anesthesia induction determined to be 0.25–0.35 mg kg^-1^. In another Phase I trial by Meier et al. (2016), CPMM, either alone or in combination with commonly used adjuncts (fentanyl, midazolam, or remifentanil), induced loss of consciousness within 7 min while maintaining spontaneous breathing, indicating that CPMM’s efficacy is not affected by preoperative or combination medications. A 2018 clinical trial by Valk et al. (2018) in healthy subjects showed that continuous infusion of CPMM at 30, 40, and 50 μg kg^-1^ min^-1^ for 30 min did not cause adrenal suppression, with plasma cortisol levels increasing by at least 200 nM after ACTH stimulation at 60 and 120 min. Although those studies further supported the feasibility of continuous CPMM infusion, however, the high incidence of involuntary muscle movements (IMM) as an adverse event (AE > 20%) of CPMM raised concerns (Meier et al., 2016). Most subjects experienced IMM at effective doses, with some requiring midazolam for relief (Valk et al., 2018; Valk et al., 2019). High-dose CPMM infusion may also be potentially associated with seizures (Valk et al., 2019). These adverse effects have introduced significant uncertainty regarding CPMM’s progression to Phase II clinical trials. Later, Valk et al. (2021) conducted a study to explore the pharmacological impact of IMM on CPMM. Their recirculation model partially reflected the relationship between IMM and the bispectral index (BIS) and modified observer’s assessment of alertness/sedation (MOAA/S). When IMM is present, BIS values reported by the BIS monitor are higher than would be expected from drug effects measured by MOAA/S due to interference in the electromyogram (EMG) frequency, thus masking the presence of an accurate enough depth of anesthesia. Individuals treated with CPMM may have BIS values higher than clinically acceptable levels, but still have sufficient brain suppressor effects (Valk et al., 2021; Dahaba, 2005). However, the above mechanism is only a conjecture based on the results of this study, so there are some limitations: it did not fully explain the cause of IMM, and the model was too simplistic to predict later drug distribution. Despite CPMM’s promising pharmacological profile, the high frequency and unpredictability of IMM have stalled further clinical trials, prompting the need for new derivatives (Valk et al., 2021).

## 5 ET-26HCl

Methoxyethyl-etomidate (ET-26) is a analogue of etomidate with a modified ester side chain (Yang et al., 2017). Hydrochloride methoxyethyl-etomidate (ET-26HCl) is formed by reacting ET-26 with hydrochloric acid in methanol, retaining the etomidate acid metabolite while reducing adrenal suppression (Wang et al., 2017a). In a 2017 study by Wang et al. (2017a), single intravenous injection of ET-26HCl in adult rats demonstrated stable hemodynamics and mild adrenal suppression, with faster recovery of spatial orientation compared to propofol, offering new possibilities for anesthesia induction in critically ill and elderly patients.

Anesthetic efficacy was measured by time of change in response to pain stimuli in rats, ET-26HCl has a lower minimum infusion rate in rats compared to CPMM (0.62 mg kg^-1^·h^-1^ vs. 0.95 mg kg^-1^·h^-1^) (Jiang et al., 2017). After continuous infusion at this rate for 60 min, the serum cortisol concentration was measured every 30 min for 4 h, and the results reflected that serum cortisol levels in rats showed significant differences from etomidate at all time points except 180 and 240 min, indicating that ET-26HCl does not significantly suppress adrenal function (Jiang et al., 2017). Based on this, previous studies designed hemorrhagic shock and septic models in rats, beagle dogs, and elderly animals (Wang et al., 2017b; Wang et al., 2017c), with results supporting the above conclusions and showing no adverse effects on myocardial function *in vivo* or *in vitro* (Liu et al., 2018). Additionally, ET-26HCl injection in rats resulted in lower levels of pro-inflammatory cytokines (IL-1β, IL-6, and IL-10) and less histological damage to the lungs and kidneys (Wang et al., 2017b), explaining its mild effects at a microscopic level. Therefore, ET-26HCl is considered to have potential clinical applications and was approved for clinical trials by the Chinese National Medical Products Administration in 2019.

Current studies have comprehensively evaluated the pharmacokinetics of ET-26HCl in preclinical settings (Zhang et al., 2019; Yu et al., 2020). Results show that the metabolites produced by human hepatocytes are similar to those from monkey, dog, rat, or mouse hepatocytes, with almost complete plasma clearance within 4 h after administration. Similarly, single and repeated dose toxicity studies in adult rats and beagle dogs showed no significant adverse effects (Zhang et al., 2020a; Zhang et al., 2020b), with mild and reversible effects on respiratory, cardiovascular, and central nervous system functions in both adult and elderly animals (Zhang et al., 2021; Chang et al., 2022). These findings support the metabolic stability and efficacy of ET-26HCl, paving the way for Phase I clinical trials in healthy volunteers.

A single-center Phase I controlled study in healthy volunteers (Yin et al., 2025) confirmed the above advantages of ET-26HCl. At a single dose of up to 2.8 mg kg^-1^, ET-26HCl showed good safety and tolerability, with a larger area under curve of plasma total cortisol (AUC_PTC_) compared to 0.3 mg kg^-1^ etomidate (614 [454] hnM vs. −932 [555] hnM), indicating milder adrenal suppression. Multiple trials (registration numbers: CTR20211150, CTR20232086, CTR20233783, CTR20233785, CTR20233669) have further explored ET-26HCl’s tolerability and anesthesia methods in patients with varying baseline conditions. Although results have not been reported, ET-26HCl’s rapid onset, dose-dependent effects, and mild cortisol suppression encourage further Phase II studies.

A Phase IIa single-center clinical investigation conducted at West China Hospital of Sichuan University (Jiang et al., 2024) established the optimal induction dose of ET-26HCl as 0.8 mg kg^-1^ through the implementation of sequential allocation and up-and-down dose-finding methodologies. In a subsequent multicenter Phase IIb study, the success rate of tracheal intubation within 7 min after induction with this dose was 100%, with MOAA/S scores ≤1 (Jiang et al., 2024). The high induction success rate, brief and reversible cortisol suppression, and no myoclonus observed in a total of 34 patients further confirmed the efficacy and safety of ET-26HCl for anesthesia induction in surgical patients. Based on these findings, multicenter, randomized, controlled Phase III clinical trials with larger sample sizes are underway (registration numbers: CTR20233036, NCT06203431) to validate the effectiveness of ET-26 in elective surgical patients.

However, some studies have shown that ET-26HCl can cause myoclonus and slow recovery of spontaneous activity (Wang et al., 2017a), as well as bradycardia and injection site pain. Although these side effects are transient and often do not require intervention, they should be noted in future trials.

## 6 Other new derivatives

In recent years, in addition to the common etomidate derivatives mentioned above, researchers have developed several new derivatives still under evaluation.

Xu et al. (2021) developed EL-0052, which has similar sedative-hypnotic effects to etomidate, with a therapeutic index of 28, significantly higher than that of etomidate. In rat studies, EL-0052 showed no statistical differences in onset time, recovery time and recovery walking time compared to etomidate. Additionally, EL-0052 had no significant effect on mean arterial pressure in beagle dogs, retaining etomidate’s hemodynamic stability. Its inhibition of cortisol biosynthesis in H259 cells was weaker than that of etomidate (IC_50_: 1050 ± 100 nM vs. 2.09 ± 0.27 nM) (Xu et al., 2021). Thus, EL-0052 retains etomidate’s favorable properties, such as effective hypnotic effects, rapid onset and recovery, and hemodynamic stability, while improving the therapeutic index and reducing adrenal suppression (Jiang et al., 2021). Further research on its adverse effects is needed.

Li Xin et al. (2023) developed ET-25-2 from etomidate, with 50% effective dose (ED_50_), median lethal dose (LD_50_), and therapeutic index of 4.15 mg kg^-1^, 39.69 mg kg^-1^, and 9.56, respectively. Although ET-25-2’s efficacy is lower than that of etomidate (therapeutic index and potency are 1.86 and 5.33 times higher, respectively), it showed no adrenal suppression in rats after ACTH administration, unlike etomidate, which still showed suppression 30 min after administration. Further investigation is required to determine the clinical applicability and pharmacological potential of ET-25-2 in well-designed experimental and clinical settings.

Deng Chaoyi et al. used molecular simulation software to analyze the docking mode of etomidate and GABA_A_ receptors. They found that the side chains adjacent to the ester bond of etomidate mainly interacted with the amino acid residues in the subunits of the GABA_A_ receptor through hydrogen bonds. Therefore, for the new compound, the modification of the side chain group can consider the inclusion of groups that can form hydrogen bonds or hydrophobic interactions with the receptor. Among the numerous computer-designed compounds, ET-11 has good solubility and further forms ET-11B by forming salts (Chaoyi, 2021). In adult SD rat models, ET-11B demonstrated rapid anesthetic onset (<1 min) and a maintenance duration comparable to etomidate (8.19 ± 3.03 min vs. 6.03 ± 1.93 min). After ACTH administration, serum corticosterone levels in rats were not significantly different from those in the saline group, indicating ET-11B have no adrenal suppression. In elderly and hemorrhagic shock rat models, ET-11B had minimal effects on hemodynamic stability. Therefore, ET-11B may be suitable for elderly, critically ill, and hemodynamically unstable patients. However, this study did not explicitly present the chemical structural formulas of ET-11 and ET-11B. Besides, it only examined single-dose administration, and further research on its pharmacological characteristics and adverse effects during continuous infusion is needed.

## 7 Conclusion

**FIGURE 1:**
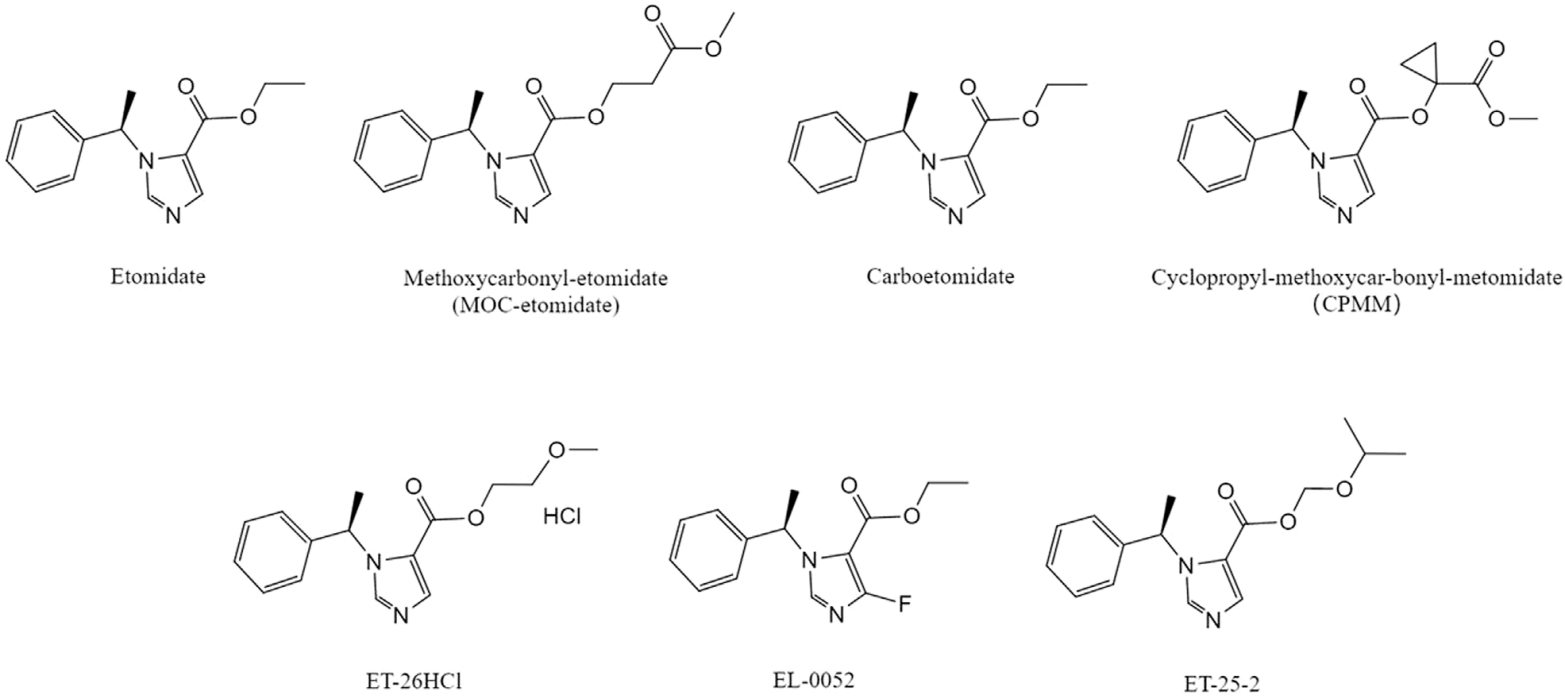
Chemical structural formula of etomidate and its derivatives .The molecular structures of MOC-etomidate (Cotten et al., 2009), carboetomidate (Shanmugasundararaj et al., 2013), CPMM (Ge et al., 2012), ET-26HCl (Wang et al., 2017a), EL-0052 (Xu et al., 2021), ET-25-2 (Li Xin et al., 2023) compared with etomidate(Source: Pubchem).

Etomidate is one of the most commonly used anaesthetic drugs, with the advantage of stable hemodynamics. However, its unavoidable adverse effects, such as adrenal suppression, POVN, injection pain, as well as the unresolved association with elevated mortality rates in critically ill patients, pose challenges to its clinical use. Currently, new etomidate derivatives have significantly reduced adrenal suppression, with CPMM and ET-26HCl showing good safety and tolerability in clinical trials. However, future development of these drugs should focus on the following: for CPMM, the mechanism of IMM should be explained at the molecular level in animal models, and more precise measurement methods should be used to determine whether it can be improved for Phase II trials. Although ET-26HCl also has side effects such as myoclonus and bradycardia, its favorable prognosis suggests that Phase III trials should proceed with close monitoring to explore its potential in critically ill patients. With ongoing research on etomidate and its derivatives, the application prospects of various etomidate derivatives are expected to broaden in the near future. The drug chemical structural formulas mentioned in the article are shown in Figure 1.

## Abbreviations

PAM, positive allosteric modulator; GABA_A_, γ-aminobutyric acid type A; PONV, postoperative nausea and vomiting; MOC-etomidate, methoxycarbonyl-etomidate; MOC-ECA, methoxycarbonyl-etomidate carboxylic acid; EEG, electroencephalogram; ACTH, adrenocorticotropic hormone; CPMM, cyclopropyl-methoxycarbonyl-metomidate; IMM, involuntary muscle movements; AE, adverse event; BIS, bispectral index; MOAA/S, modified observer’s assessment of alertness/sedation; EMG, electromyogram; ET-26, methoxyethyl-etomidate; ET-26HCl, hydrochloride methoxyethyl-etomidate; AUC_PTC_, area under curve of plasma total cortisol; ED_50_, 50% effective dose; LD_50_, median lethal dose.
